# Supplementary material for: A comparative anatomy of protein crystals: lessons from the automatic processing of 56 000 samples
Source: IUCrJ. 2019 Jul 10;6(Pt 5):822–31. doi: 10.1107/S2052252519008017 (PMC6760449; doi:10.1107/S2052252519008017)
Supplement: Supplementary file 1 [file m-06-00822-sup1.pdf]

IUCrJ

Volume 6 (2019)

Supporting information for article:

A comparative anatomy of protein crystals: lessons from the automatic processing of 56 000 samples

Olof Svensson, Maciej Gilski, Didier Nurizzo and Matthew W. Bowler

Table S1 Verification of calculated molecular weights compared to known samples.

Acronym	Actual MW (kDa)	Calculated MW (kDa)
Lyso	14.2	12
RICK	34.85	48
CRU	41.31	43
Erk2	42.24	41
DPP	162	200
TbrATP	922.6	800
GroEL	910	700

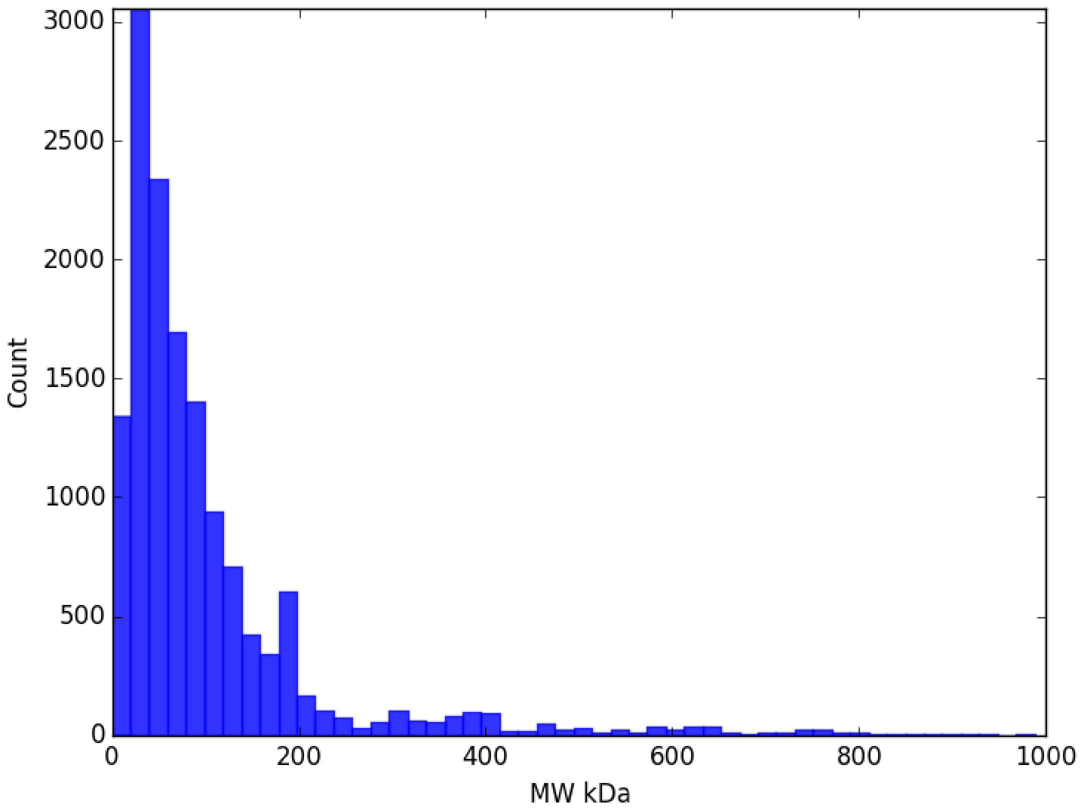

Figure S1 Histogram of molecular weights calculated from samples processed on MASSIF-1.
